# The psychological burden of volunteers in psychosocial emergency care – a qualitative interview study

**DOI:** 10.1007/s12144-021-01382-7

**Published:** 2021-02-09

**Authors:** Anja Greinacher, Anna Cranz, Julia Jenzer, Alexander Nikendei, Renate Kottke, Jürgen Wiesbeck, Hans-Christoph Friederich, Christoph Nikendei

**Affiliations:** 1 Center for Psychosocial Medicine, Department of General Internal Medicine and Psychosomatics, University Hospital Heidelberg, Thibautstrasse 4, 69115 Heidelberg, Germany; 2 Institute of Psychology, Heidelberg University, Heidelberg, Germany; 3 German Red Cross, Rescue Service Bodensee-Oberschwaben, Ravensburg, Germany; 4 German Red Cross, Baden-Wuerttemberg Regional Association, Stuttgart, Germany

**Keywords:** Psychosocial emergency care, First responders, Secondary traumatization, Psychological burden, Qualitative research, Interviews

## Abstract

Psychosocial emergency care personnel provide voluntary psychological support directly after potentially traumatic events. During emergency responses, they experience challenging situations. However, previous quantitative studies suggest that the psychological burden of psychosocial emergency care personnel does not exceed that of the general population. This study aimed to obtain an in-depth analysis of the volunteers’ psychological reactions and resources regarding emergency responses. 36 psychosocial emergency care volunteers (12 pre-training, 12 post-training, 12 experienced) were invited to participate in semi-structured interviews. The volunteers were selected from previous cross-sectional and longitudinal studies on secondary traumatization in psychosocial emergency care volunteers. A qualitative content analysis of the transcribed interviews was performed following the principles of summary and inductive category development. We identified 845 codes which we summarized in three overarching categories: (I) reactions to emergency responses, (II) psychosocial emergency care work related resources and (III) experiences and changes in life perspective related to working in psychosocial emergency care. The volunteers described both emotional and physical reactions to emergency responses. While they perceived social support as a key coping resource and reported a greater appreciation of their own lives and their families due to their work, many volunteers also felt increased concern that something could happen to them. The volunteers’ reactions and symptoms are reasonable responses to stress and not indicative of serious impairment. Nevertheless, emergency responses are both emotionally and physically challenging. Volunteers should be carefully selected, receive regular supervision and determine the frequency of emergency responses.

## Background

First responders are the first on the scene after accidents and other disasters, attending to people, property, and the environment (Prati & Pietrantoni, 2010). Psychosocial emergency care personnel are a subset of first responders. Their task is to provide short-term psychological support to others immediately after potentially traumatic events. Their objective is to prevent immediate or chronic maladaptive psychosocial stress reactions in those affected (Beerlage, 2009). Common emergency responses include assisting the injured, supporting those who have received the news of an imminent death, supporting families after the death of a child or infant, or accompanying the police in delivering a death notice. Furthermore, psychosocial emergency care plays an important role in the psychological care of infected patients, their relatives, and hospital staff during the current COVID 19 pandemic (Deffner, Hierundar, Arndt, & Hinzmann, 2020; Rentrop, Schneider, Bäuerle, Junne, Dörrie, Skoda, Schedlowski, Mallmann, Benecke, Kohler, Gerigk, Teigelack, Emler, Scherbaum, Gradl-Dietsch, Scheer, & Teufel, 2020).

While volunteers are confronted with psychologically challenging situations, they do not usually experience life-threatening situations themselves during emergency operations. Based on current guidelines, psychosocial emergency care personnel should not arrive until after the event and, hence, should no longer experience others during life-threatening situations. Before the introduction of the current version of the Diagnostic and Statistical Manual of Mental Disorders (DSM-5; American Psychiatric American Psychiatric Association, 2013), both circumstances were central diagnostic criteria for primary trauma diagnosis. In the DSM-5, the concept of the cause of traumatization was extended to the effect that even a report of a traumatic event can lead to post-traumatic symptoms and trauma-related diseases. Volunteers in psychosocial emergency care regularly listen to the experiences of those affected. This can be emotionally challenging, overburden the volunteers’ coping skills, lead to high levels of stress, and cause psychological and physical discomfort.

Already in the 1980s, Figley (1999) described the phenomenon of secondary traumatization ‘as the stress deriving from helping others who are suffering or who have been traumatized’. The symptoms can be similar to post-traumatic stress disorder (Reinhard and Maercker, 2004). According to the DSM-5 (American Psychiatric Association, 2013), the following criteria must be met for the diagnosis of secondary trauma: intrusive memories, avoidance of thoughts, feelings, or stimuli that remind one of the traumatic experience; negative changes in thoughts and mood, e.g. alienation, negative trauma-associated emotions; and changes in arousal level and responsiveness, e.g. hypervigilance, concentration difficulties, sleep disorders. A specific form of secondary traumatization is vicarious traumatization, which describes a change in cognitions in the sense of a negative view of the world. Vicarious traumatization is an established consequence of working with traumatized people (Pearlman and Saakvitne, 1995). Depressive and anxiety symptoms, depersonalization, emotional exhaustion, obsessive compulsive symptoms (Tehrani, 2016), as well as changes in professionals’ views of people and the world can also occur (Pearlman and Saakvitne, 1995). In addition to hearing traumatic experiences, other factors also influence the development of secondary traumatization, including empathy (Albeck, 1994), emotional contagion (Hatfield, Carpenter, & Rapson, 2014), and genetic factors (Kellermann, 2013).

Other factors, so-called resilience factors, seem to have a protective function. Zautra, Hall, & Murray (2010) defined resilience as ‘an outcome of successful adaption to adversity’. Rather than exclusively describing the absence of symptoms, it is seen as an independent, dynamic process (Keyes, 2002). It is important to distinguish that there are work-related and individual factors that can protect against trauma sequelae (Olff, Amstadter, Armour, Birkeland, Bui, Cloitre, Ehlers, Ford, Greene, Hansen, Lanius, Roberts, Rosner, & Thoresen, 2019). Work-related protective factors can be pre-employment selection, stress management training, and early intervention (Skogstad, Skorstad, Lie, Conradi, Heir, & Weisæth, 2013). In current literature, social support is one of the most studied resilience factors among first responders (Guilaran, de Terte, Kaniasty, & Stephens, 2018; Prati & Pietrantoni, 2010). Protective functions have also been demonstrated for other factors, such as mindfulness, internal control, self-efficacy, and engagement (Greinacher, Derreza-Greeven, Herzog, & Nikendei, 2019a). There is evidence that resilience factors for work-related trauma can be trained and improved, at least over a period of time (Anderson, Vaughan, & Mills, 2017; Vaughan, Stoliker, & Anderson, 2020).

A previous quantitative study showed no increased psychological burden among volunteers in psychosocial emergency care. Volunteers reported fewer symptoms of secondary traumatization than other professionals working with comparable clientele. The burden of anxiety and depression symptoms was comparable to that of the normal population (Greinacher, Nikendei, Kottke, Wiesbeck, Herzog, & Nikendei, 2019b). Nevertheless, several authors have pointed out that the psychological burden of first responders might be underestimated owing to positive response bias (Greinacher et al., 2019a). For this reason, we conducted interviews with volunteers in order to gain a deeper insight into the stress and resilience factors in this specific group of first responders.

## Methods

### Psychosocial Emergency Care in Germany

Intensive training is required for people wishing to volunteer in emergency psychosocial care once they have been selected. Training itself is highly practice-oriented, whereby case discussions and the use of role play are significant elements. In three weekend seminars over a period of 6 months, the volunteers are trained in basic psychosocial emergency care (e.g., emergency organizations and logistics), legal frameworks, essential psychological and psychiatric knowledge, and crisis communication. Furthermore, the program also covers more intimate topics including dealing with death personally and the importance of self-care. Between the second and third weekend, the volunteers begin to shadow experienced psychosocial emergency care personnel during their emergency responses. Psychosocial emergency care is an integral part of the rescue chain. Emergency centers automatically alert psychosocial emergency staff in emergency situations where psychosocial support may be needed (e.g. suicide, child crib death, accidents; Nikendei, 2012). Following their training, each volunteer decides individually how many hours they would like to work as a volunteer. Volunteers are on call around the clock during their shifts. Generally, volunteers work in pairs on emergency responses allowing them to communicate during these and to discuss responses afterwards. Following the emergency responses, volunteers are required to debrief with their supervisor and to write a report.

### Participants and Procedure

The study was conducted between March 2017 and April 2018. We invited all Red Cross psychosocial emergency care volunteers who had participated in our quantitative cross-sectional (Greinacher et al., 2019b) and longitudinal studies (Greinacher, Nikendei, Kottke, Wiesbeck, Herzog, Friederich, & Nikendei, 2020) investigating secondary traumatic stress and psychological burden in psychosocial emergency care volunteers to participate in this interview study. We included the first 12 consenting volunteers from each group. In total, 36 volunteers (*n* = 27 female, age: *M* = 50.20, *SD* = 12.40) participated in telephone interviews; of which 12 were novice volunteers just starting their training who had not yet participated in any emergency responses (PRE-NOV), a further 12 novice volunteers who had just completed their training and had accompanied experienced volunteers to emergency responses (POST-NOV), and 12 experienced volunteers (EXP, ‘experts’) who had completed their training and had participated in regular emergency responses for M = 9 years, range 3–17.

### Development of Interview Guideline

After an in-depth literature review, a team of experts (*N* = 4; 2 female, 2 male, all of whom experienced in psychotherapy training and research) developed the interview questions and hypotheses. The COREQ checklist was used as a reference that provides guidance to researchers to conduct and report qualitative research, e.g. methodological orientation and theory (Tong, Sainsbury, & Craig, 2007). The semi-standardised interview manual (Knox & Burkard, 2009) contains open leading questions followed by clarifying questions.

In accordance with Helfferich (2011), participants were interviewed on their experiences during and after the emergency responses as well as how they dealt with these experiences and their perceived support. Pre-training novices were asked about their expected burdens and possible effects. Following the semi-standardised interview guideline (Tables 1 and 2), the individual interviews were conducted by a trained, female interviewer (JJ) who was supervised by an experienced colleague (CN) who had conducted similar studies. All interviews were digitally recorded and lasted between 15 (PRE-NOV) and 30 min (POST-NOV, EXP).

**Table 1:** Semi-standardised interview guideline for pre-training novices

Leading question	Further questions	Notes
***Question 1: Expectations***		
What did you expect from psychosocial emergency care beforehand? In other words, what were your ideas about the job before you started training?	What are you looking forward to? What makes you curious? What challenges do you expect? What fears or concerns do you have before your first assignment?	
***Question 2: Anticipated burdens and implications***		
How do you expect emergency response work to affect you personally?	Impact on mental health? Impact on attitudes / values? Positive effects?	Impact on your stress-perception? Effect on your ability to deal with difficult or dramatic situations

**Table 2:** Semi-standardised interview guideline for post-training novices and experts

Leading question	Further questions	Notes
***Question 1: Subjective experience during the emergency responses***		
In your psychosocial emergency care related work, what particular experiences have you had - in general and personally?	Have the impressions changed over time? How did you personally experience the responses on site? → What feelings did you feel during the responses? → Were there difficult situations? → Were there good moments? Were there any effects on your mental well-being? Effects on attitudes / values? Positive effects?	Was it distressing? Did it make you sad? Or didn’t you “feel” much?
***Question 2: Subjective experience after the responses***		
With which thoughts or feelings do you usually leave emergency responses?		
Do you think about your psychosocial emergency care response afterwards?	What do you think about the most?	
***Question 3: Mental burden***		
There is the phenomenon that people who are in close contact with very stressed or traumatized people or those affected can also be psychologically burdened. Would you be able to say the same about yourself?	Insomnia, restlessness, avoidance, re-experiencing, depressive symptoms, anxiety?	
Have your own values changed as a result of your previous emergency responses?		Vicarious traumatization
***Question 4: Processing of experiences***		
How do you manage the experiences you have during your psychosocial emergency responses?	Have you talked to someone? Did you discuss issues that you found distressing?	
What would you need to do to manage the experiences even better?	Organizationally? In training? From superiors? Improvement of specific personal skills?	
***Question 5: Interventions***		
Do you feel well supported in your work in emergency care?	What support do you get? What is particularly important to you in this context? Do you feel valued for your work?	
What kind of support would you like to receive?	Organizationally? In training? From superiors? Improvement of specific personal skills?	
***Question 6: Overall conclusion***		
What conclusion would you draw from your experience in psychosocial emergency care so far?		

### Ethics Approval and Consent to Participate

Prior to the realization of the study, it was approved by the ethics committee of the University Hospital Heidelberg (ethics application no. S-139/2016). All volunteers that participated in the study provided informed written consent and were free to withdraw their participation without any disadvantage. The study was conducted in accordance with the Declaration of Helsinki (most recent version: Fortaleza, Brazil, 2013).

### Qualitative Content Analysis and Quantitative Statistical Analysis

After verbatim transcription of the 36 interview audio files, we performed a qualitative content analysis following the principles of summary and inductive category development (Mayring, 2000). First, we conducted an open coding of all interview transcriptions line by line. In detail, single or few sentences were identified as a code, representing the most elemental unit of meaning (Strauss & Corbin, 1998). Next, the codes were summarized into abstracted relevant themes for each participant, using the software “MAXQDA” (VERBI Software, 2018). As themes were recurrent among different participants, they were compared and adapted until a number of relevant themes for all participants could be defined. Two independent analyzers (JJ, AG) assigned the respective codes to specific themes and subsequently discussed them to reach consensus (investigator triangulation). In a final step, themes similar in content were grouped into relevant categories. Descriptive socio-demographic data were managed with the software package SPSS (IBM SPSS Statistics 20) and presented as mean, standard deviation, and range.

## Results

### Main Categories and Themes

The qualitative analysis of the interviews revealed 845 single codes. These codes were summarized and inductive themes were formed. The three main categories with associated themes are described in detail below. The number of codes per category and theme is shown in parentheses; a distinction was made between volunteers before training (PRE-NOV), volunteers after training (POST-NOV), and experienced volunteers (EXP, ‘experts’). A more detailed description of the topics can be found in Table 3. Illustrative quotations for main categories and themes are listed in Table 4.

**Table 3:** Qualitative analysis results: The three main categories with associated themes with number of single codes shown in parentheses

	**1. Reactions related to emergency response work (232)**	
	**T2: Post-training novices (120)**	**T3: Experts (112)**
	T2–1.1 Thoughts regarding emergency responses (35)	T3–1.1 Emotional reactions (29)
	T2–1.2 Emotional reactions (28)	T3–1.2 Thoughts regarding emergency responses (27)
	T2–1.3 Do not let it get to you (17)	T3–1.3 Undefined symptoms (23)
	T2–1.4 Emergency responses encourages reflections on life (13)	T3–1.4 Do not let it get to you (11)
	T2–1.5 Compassion, not pity (12)	T3–1.5 Emergency responses encourages reflections on life (9)
	T2–1.6 Physical reactions (7)	T3–1.6 Physical reactions (5)
	T2–1.7 Undefined symptoms (6)	T3–1.7 Compassion, not pity (4)
	T2–1.8 Familiy feedback on personal health (2)	T3–1.8 Images of emergency responses before the mind’s eye (4)
**2. Expected resources related to emergency response work (18)**	**2. Resources related to emergency response work (158)**	
**T1: Pre-training novices (18)**	**T2: Post-training novices (65)**	**T3: Experts (93)**
T1–2.1 Peer support (team leadership, team evenings, supervision) (5)	T2–2.1 Peer support (team leadership, team evenings, supervision) (25)	T3–2.1 Peer support (team leadership, team evenings, supervision) (30)
T1–2.2 Engaging in recreational activities (4)	T2–2.2 Support by engaging with team partners during and after the response (18)	T3–2.2 Support by engaging with team partners during and after the response (19)
T1–2.3 Talking about the emergency response (4)	T2–2.3 Engaging in recreational activities (6)	T3–2.3 Talking to family and friends (9)
T1–2.2 Rituals (2)	T2–2.4 Talking to family and friends (6)	T3–2.4 Writing of emergency response reports (9)
T1–2.5 Support by engaging with team partners during and after the responses (2)	T2–2.5 Wearing certain clothes (4)	T3–2.5 Wearing certain clothes (6)
T1–2.6 Learning skills (1)	T2–2.6 Thinking about the emergency responses (2)	T3–2.6 Engaging in recreational activities (6)
	T2–2.7 Writing of emergency response reports (2)	T3–2.7 Only going on emergency response when feeling stable (6)
	T2–2.8 Personal time after emergency responses (2)	T3–2.8 Thinking about the emergency response (3)
	T2–2.1 Peer support (team leadership, team evenings, supervision) (25)	T3–2.9 Personal time after emergency responses (3)
		T3–2.10 Rituals (2)
**3. Expected experiences and changes related to emergency response work (31)**	**3. Experience and changes related to emergency response work (125)**	
**T1:**: **Pre-training novices (31)**	**T2: Post-training novices (50)**	**T3: Experts (75)**
T1–3.1 Personal growth (10)	T2–3.1 Increased satisfaction and contentment with one’s life (20)	T3–3.1 Increased satisfaction and contentment with one’s life (22)
T1–3.2 Improved satisfaction and contentment with one’s own life (9)	T2–3.2 Personal growth (12)	T3–3.2 Appreciation and gratitude shown by those concerned (17)
T1–3.3 The feeling of “doing something good” (7)	T2–3.3 Appreciation and gratitude shown by those concerned (11)	T3–3.3 Experienced meaningfulness of the work (16)
T1–3.4 Appreciation and gratitude shown by those concerned (3)	T2–3.4 Psychosocial emergency care team spirit (7)	T3–3.4 Insights into unfamiliar circumstances and unusual situations (14)
T1–3.5 Increased concern about accidents in one’s family (2)		T3–3.5 Psychosocial emergency care team spirit (6)

**Table 4:** Quotations for main categories and themes

**1. Reactions related to emergency response work (232)**	
**T2: Post-training novices (120)**	
**T2–1.1** Thoughts regarding emergency responses (35) •"Certain things that get under your skin or open questions that you need to discuss, for example in supervision sessions. •"Things that come up again and again because you’ve looked it up on media and think: what will happen to that family? That’s the kind of thing where I realize: Yes, I won’t forget that case, I’ll keep it with me for a while.”	
**T2–1.2** Emotional reactions (28) •„It was simply sadness and bewilderment and also helplessness after such a situation.” • “I sat at the table and suddenly started to cry.”	
**T2–1.3** Do not let it get to you (17) • “Because there are other things on my mind, especially during the responses, there were lots of other things, you’d have to organize something, there was no time to feel inside yourself at that moment during the response.”	
**T2–1.4** Emergency responses encourage reflections on life (13) • “Yes, where one then also thinks, where I can draw parallels to my own life.”	
**T2–1.5** Compassion, not pity (12) • “Well, I sympathize, but I don’t suffer anymore.” • “I feel it, but I don’t grieve. I deal with it rather well.”	
**T2–1.6** Physical reactions (7) • “But then I was surprised that every time I passed the place where this happened, I’d feel a little jitter.” • “...that one’s blood pressure or adrenaline goes up, it calms down after a few days.”	
**T2–1.7** Undefined symptoms (6) • “It gets under your skin.”	
**T2–1.8** Familiy Feedback on personal health (2) • “...our eldest daughter said: Well mom, I think it’s time you got some supervision.”	
**T3: Experts (112)**	
**T3–3.1** Emotional reactions (29) •"I’m very emotionally involved. I also have responses where I have to go out and cry for a minute sometimes.” •”...the sadness, you feel it seep from the family, that’s obvious.”	
**T3–3.2** Thoughts regarding emergency responses (27) •"I often think about the bereaved: What will they do, how do they cope?”	
**T3–3.3** Undefined symptoms (23) •"No, that’s pretty much what got me down, I’ve been struggling with that for a while too.” •„It affects me.“	
**T3–3.4** Do not let it get to you (11) •"When out in the field, I sort of shut off my own emotions.” •"Sometimes I feel a wave of helplessness and I have to wake up the spirit inside me: “Don’t get involved in this. I try to flip the inner switch.”	
**T3–3.5** Emergency responses encourage reflections on life (9) •”...when my children were in a vulnerable age, in adolescence, and you got into accidents or something, that was always very bad. Sometimes it was really hard to keep your distance because you’d just identify with it.”	
**T3–3.6** Physical reactions (5) •"I know that the day after that I’m mentally and physically exhausted.”	
**T3–3.7** Compassion, not pity (4) • “I realize, of course, that I need a certain distance to see, it’s not mine, I don’t want to go all the way in there, I don’t want that, I’m not a relative.”	
**T3–3.8** Images of emergency responses before the mind’s eye (4) •"And I have an inner image of the scene, it no longer weighs on me, but I still see the image before my inner eye.”	
**2. (Expected) Resources related to emergency response work (176)**	
**T1: Pre-training novices (18)**	
**T1–2.1** Peer support (team leadership, team evenings, supervision) (5) •"This family-like group that just meets every month to talk, that’s a total network for me, where I know, [...], I’ll be supported there.”	
**T1–2.2** Engaging in recreational activities (4) •"For example, it helps me to paint pictures, to be creative in some way, that helps me.”	
**T1–2.3** Talking about the emergency response (4) •“...talking to someone about it...”	
**T1–2.4** Rituals (2) •"Yeah, that’s where we got the advice on how to ‘take off your jacket’ after a response.”	
**T1–2.5** Support by engaging with team partner during and after the responses (2) •"For me it is important that we do these responses together, that is, that there are actually two of us. I think you’d have more problems if you did it alone.”	
**T1–2.6** Learning skills (1) •"We have been trained and have learn skills.”	
**T2: Post-training novices (65)**	
**T2–2.1** Peer support (team leadership, team evenings, supervision) (25) • “The supervisor really took the whole hour for this and asked a lot of questions and there was a lot of discussion in the group and that helped me a lot.”	
**T2–2.2** Support by engaging with team partner during and after the responses (18) •"There is also an agreement among us that we have the possibility to call our colleagues day and night, if something won’t let go of you.” •"If the situation and the time of day allow, we’ll also go for a coffee, that’s also very nice to close things up.”	
**T2–2.3** Engaging in recreational activities (6) •"I like walking my dog [afterwards].”	
**T2–2.4** Talking to family and friends (6) •"Just talk to my family, that my wife listens to me when I come back from a response and we talk about it a bit.”	
**T2–2.5** Wearing certain clothes (4) •"And how much inner strength that stupid blue Red Cross anorak gives me. Because, we wear it to identify ourselves, and then you realize immediately: I’m on duty now, that’s not my personal story, but I’m here for the affected family.”	
**T2–2.6** Thinking about the emergency response (2) •”… to process the whole thing for myself, to go through the response again in my mind and then to say ‘goodbye’ to the situations in my mind.”	
**T2–2.7** Writing the emergency response report (2) •"Writing the emergency response reports is also important after a high-stress situation. When I’ve written the protocol, I’ve put the operation behind me and I’ve put the operation behind me.”	
**T2–2.8** Personal/ „Me“ time after the emergency response (2) •"To be alone and not have to talk….”	
**T3: Experts (93)**	
**T3–2.1** Peer support (team leadership, team evenings, supervision) (30) •"In terms of processing the responses, we are actually well looked after by the team: we have our supervision, we have our regular meetings with the team, our team management actively asks how you are doing, and you know that you can call them at any time.”	
**T3–2.2** Support by engaging with team partner during and after the responses (19) • “After a response mission, I always try, if it is somehow possible, to sit down together afterwards and talk to each other: How are you, how did you feel about it? That’s very important to me.”	
**T3–2.3** Talking to family and friends (9) •- “...I find talking to my husband very helpful.”	
**T3–2.4** Writing the emergency response report (9) •- “...then I’ll write my report, and then I’m done.”	
**T3–2.5** Wearing certain clothes (6) •"I will not go on any response mission without a uniform. I come home, hang the uniform on the hanger, and then it’s done, then it’s gone. It’s a ritual. I also have certain shoes and socks which I wear.”	
**T3–2.6** Engaging in recreational activities (6) •"Music helps me a lot. I’ve been singing in a choir for almost 40 years now.”	
**T3–2.7** Only going on emergency response when feeling stable (6) •”...because we only go into action when we feel good. The most important thing in response missions is me; if I don’t feel good, I can’t help anyone. So you can refuse to go, without a giving reason, I think that’s excellent.”	
**T3–2.8** Thinking about the emergency response (3) •"I review everything and if there is something that makes me feel uncertain, I take it up and investigate: Why do I feel insecure?”	
**T3–2.9** Personal/ „Me “time after the emergency response (3) •"I’m so the type of guy, I like to hike. And then I have to see that I’m alone somewhere, that I can let my thoughts flow. It helps me.”	
**T3–2.10** Rituals (2) •"So I come home, I don’t say hello to anyone, I change my clothes, and all the dirty laundry goes straight in to the washing machine.”	
**3.(Expected) experiences and changes related to emergency response work (156)**	
**T1: Pre-training novices (31)**	
**T1–3.1** Personal growth (10) •"I think it’s likely to be good for your self-esteem, I would imagine.”	
**T1–3.2** Improved satisfaction and contentment with one’s own life (9) •"I think I’m becoming more humble and grateful for my own life.”	
**T1–3.3** The feeling of “doing something good” (7) •"And I think it’s a good feeling to know that you can support people when they are in need. And maybe, even if it’s just a little thing, maybe you are able to do something good for people.”	
**T1–3.4** Appreciation and gratitude shown by those concerned (3) •”...that people are grateful that someone is there.”	
**T1–3.5** Increased concern about accidents in one’s family (2) •"Then you’re even more afraid for your children. If I am called to an accident now where young people are involved [...], then I would naturally be all the more afraid for my children when they are on the road.”	
**T2: Post-training novices (50)**	
**T2–3.1** Improved satisfaction and contentment with one’s own life (20) •"Of course, you do appreciate being healthy more. You can enjoy some things more or appreciate them more.” • “Being with your family or things like that has taken on a whole new meaning for me.”	
**T2–3.2** Personal growth (12) •"It encouraged me to be more open with people, ask better questions, I think.”	
**T2–3.3** Appreciation and gratitude shown by those concerned (11) •"In my practical experience so far, I can also say that it works and people show their appreciation afterwards and through their gratitude I also get back the, how shall I put it, satisfaction in my voluntary work.”	
**T2–3.4** Psychosocial emergency care team spirit (7) •"We’re happy to see each other – we don’t meet up for birthdays or anything private - but we’re happy to see each other when we get together and that’s really nice.”	
**T3: Experts (75)**	
**T3–3.1** Improved satisfaction and contentment with one’s own life (22) •"I have become much more humble. And the satisfaction. And “What is important in life?”	
**T3–3.2** Appreciation and gratitude shown by those concerned (17) •”...if you get a thank you or something from people in a situation like that, that’s the greatest appreciation you can get.”	
**T3–3.3** Experienced meaningfulness of the work (16) •"And after every response mission, when you leave, you really feel like: I was able to help here. And this makes up for a lot.”	
**T3–3.4** Insights into unfamiliar circumstances and unusual situations (14) •"I’m just always surprised how versatile this job is, the situations you get into, it’s incredibly impressive.”	
**T3–3.5** Psychosocial emergency care team spirit (6) •"The team spirit of the group, i.e. that you meet so regularly with the team and that you can talk about all kinds of things in the team and it’s not just about the work and the last emergency responses. That we have created a sense of familiarity through this. And that’s very important to me.”	

### Category I. Reactions to Emergency Responses (232)

*Shared participant group themes.* Post-training novices and experts reported largely identical reactions to emergency responses. Frequently, volunteers responded with primarily emotional reactions (POST-NOV: 28; EXP: 29). They described feelings of sadness especially, and some participants reported having cried. Furthermore, feelings of helplessness, anger, and incomprehension were reported. However, some volunteers also described experiencing positive feelings regarding their own lives and health status. Volunteers reported regularly remaining mentally preoccupied with the emergency responses afterwards (POST-NOV: 35; EXP: 27). In particular, the experienced volunteers reported ruminating thoughts after emergency responses: while some participants mentally reran the response again and again, others thought about the casualties, their relatives, and/or the survivors from time to time. Physical reactions, such as physical exhaustion or palpitations, were also described (POST-NOV: 7; EXP: 5). Participants also used metaphors to describe certain emergency responses and their reactions (POST-NOV: 6, EXP: 23). Experienced volunteers noticed an increase of their thoughts about their private lives due to the emergency responses: they were reminded of their own family when, for instance, affected children were of same age as their own. Consequently, they described to either feel increased worry that something might happen to their relatives or feeling grateful that their relatives were healthy and unhurt. Some participants described having changed things in their lives, e.g. taking out nursing care insurance (POST-NOV: 13; EXP: 9). However, the volunteers also described distancing behavior regarding their emergency response work feeling that although they wanted to be empathic during the emergency responses, but they did not want to suffer personally (POST-NOV: 12; EXP: 4) by letting the situations get too close to them (POST-NOV: 17, EXP: 11). Some participants described trying to switch their own emotions ‘off’ in order to keep an inner distance.

*Differences in participant group themes.* Post-training novices explained how they had only noticed feeling stressed following an emergency response after a relative had asked them about it (POST-NOV: 2). The experts, who had already accompanied multiple emergency responses, reported seeing images the emergency responses appearing before their mind’s eye (EXP: 4).

### Category II. (Expected) Psychosocial Emergency Care Work Related Resources (176)

#### Shared Participant Group Themes

Pre-training novices expected and the two other participant groups experienced social and professional support (PRE-NOV: 5, POST-NOV: 25, EXP: 30), team leadership, joint team evenings, and supervision (internal and external) as key work-related resources. They felt particularly supported by their volunteer partners, with whom they worked emergency response and shared experiences afterwards (PRE-NOV: 2, POST-NOV: 18, EXP: 19). Conversations with non-first-responders, e.g. friends and family, were also perceived as supportive (PRE-NOV: 4, POST-NOV: 6, EXP: 9). In addition, volunteers reported that they often consciously engaged in positive, self-soothing activities, including cooking, crafting, listening to or making music, or engaging with their pets, after emergency responses (PRE-NOV: 4, POST-NOV: 6, EXP: 6).

#### Differences in Participant Group Themes

Post-training novices and experts reported that wearing their uniforms was helpful during emergency responses (POST-NOV: 4, EXP: 6) feeling that this helped them to empathize and identify with their professional role. Furthermore, the uniforms were seen to facilitate leaving the day’s events behind them mentally by physically changing from their uniform to their everyday live attire. In addition, pre-training novices and experts mentioned further work-related rituals (PRE-NOV: 2, EXP: 2), e.g. wearing special socks during or showering after an emergency responses in order to underline their return to everyday life afterwards. After emergency responses, the experts reported taking some time alone (POST-NOV: 2, EXP: 3) to reflect the emergency response alone (POST-NOV: 2, EXP: 3) and to write the obligatory emergency response reports (POST-NOV: 2, EXP: 9). Pre-training novices expected their training to help them cope with the emergency responses (PRE-NOV: 1), while the experts stressed the importance of only participating in emergency response when they themselves felt mentally stable enough (EXP: 6).

### Category III. (Expected) Experiences and Changes in Life Perspective Related to Working in Psychosocial Emergency Care (156)

#### Shared Participant Group Themes

All three groups of participants reported an increased satisfaction as well as contentment in regard to their own lives as a result of working in psychosocial emergency care (PRE-NOV: 9, POST-NOV: 20, EXP: 22). The appreciation and gratitude of those affected was also important to all volunteers as a reward for their efforts (PRE-NOV: 3, POST-NOV: 11, EXP: 17).

#### Differences in Participant Group Themes

Pre- and post-training novices anticipated that their volunteer work would result in positive personal development (PRE-NOV: 10, POST-NOV: 12). Pre-training novices and experts emphasized the experienced meaningfulness of their work (PRE-NOV: 7, EXP: 16). Post-training novices and experts pointed out that team coherence and team spirit was very important to them (POST-NOV: 7, EXP: 6). While pre-training novices experienced a heightened concern over accidents in their personal environment (PRE-NOV: 2), the experts emphasized the multifacetedness of their work. The experts stated that emergency responses allowed them to gain greater insight into different life circumstances and extreme psychosocial situations (EXP: 14). This insight sometimes confronted the volunteers with foreign perspectives leading them to reflect on their own moral views.

## Discussion

For this study, we interviewed 12 pre-training novice volunteers who had not yet been involved in emergency responses, 12 post-training novice volunteers shadowing experienced volunteers during emergency responses, and 12 experienced (expert) volunteers who had regularly participated emergency responses for at least 3 years in psychosocial emergency care about their experiences and personal reactions to emergency responses, their resources as volunteers and changes in their lives resulting from their volunteer work.

### Secondary Traumatization

The volunteers who had participated in emergency responses reported experiencing both emotional (e.g. sadness, helplessness and anger) and physical reactions (e.g. physical exhaustion or palpitations) related to their emergency response work and often expressed these reactions with the help of metaphors in the interviews. They reported experiencing reactions either during or immediately after the emergency response and that these reactions usually resolved rapidly. However, some reported re-experiencing individual reactions (e.g. increased heart rate) when reminded of specific emergency responses later. However, the reactions described would appear to be appropriate responses to severe stress and therefore *do not* necessarily indicate any serious impairment as may occur in acute stress disorders (World Health Organization (WHO) 1993). These results are consistent with previous research, e.g. parole officers also reported experiencing incident-related emotional and somatic reactions (Severson & Pettus-Davis, 2013). In addition, psychosocial emergency care volunteers also had ruminative thoughts about the emergency response and expressed concerns and fears regarding their personal lives as a result of their emergency response activities, e.g. they sometimes worried that something might happen to themselves or their family. Some volunteers consequently tried to change certain aspects of their lives, e.g., taking out long-term care insurance. At extreme levels, such a process of anxious reflection can result in a negative worldview which might be understood as a symptom of vicarious traumatization (Pearlman & Saakvitne, 1995).

A few experienced volunteers also reported seeing images of emergency response before their mind’s eye. Intrusive images or memories of a stressful event are a hallmark of post-traumatic stress disorder. However, the images’ intrusive quality was described as low. Moreover, a single symptom is not sufficient to warrant a secondary trauma diagnosis. In line with these findings, quantitative studies assessing psychosocial emergency care volunteers’ psychological burden (Greinacher et al., 2019b, 2020) revealed their levels of secondary and primary traumatization to be below cut-off scores. The emergency care volunteers’ level of depressive symptoms was comparable to representative norm samples, while they showed anxiety levels below norm sample values. Analogous findings regarding secondary trauma were shown in a systematic review of studies regarding the psychological burden on first responders in general (Greinacher et al., 2019a). Evidence suggests that volunteers in psychosocial emergency care working as first responders generally respond to emergency responses with emotional and physical reactions and cognitive changes (vicarious traumatization). However, they do not show clinical symptoms of secondary traumatization.

### Resilience Factors

In the conducted interviews, volunteers shared their resources to improve coping with their emergency response associated stress. They named the support of colleagues, especially their volunteer partners, professional supervision, as well as non-expert supporters (e.g. family) to be very helpful. Hence, intra-organizational peer support seems to be as important as a supportive social environment outside the volunteer work. Previous qualitative studies in first responders have also identified social support as a major resilience factor (Burns, Morley, Bradshaw, & Domene, 2008; Powell, Cassematis, Benson, Smallbone, & Wortley, 2014). Furthermore, social support was shown to be negatively associated with psychological burden in a quantitative study assessing first responders in general (Greinacher et al., 2019a). Providing volunteers with social and organizational support services should be an integral part of organizations delivering emergency mental health care. During interviews, volunteers shared process promoting social support within their organization. Volunteers are always deployed in pairs facilitating peri-response communication. Responses are routinely followed by a mandatory debriefing with the supervisor and regular team supervision sessions are offered. Furthermore, regular team evenings are organized for all volunteers to encourage communication and the exchange of personal response-related experiences. Supervision sessions hold a key role here as further coping resources, previously identified in literature such as active problem solving, self-reflection and competence building, can be developed and discussed here. (Wyche, Pfefferbaum, Pfefferbaum, Norris, Wisnieski, & Younger, 2011).

Volunteers reported an additional coping strategy in that they tried to distance themselves from the emergency response during and after the emergency response. They reported trying to avoid letting their experiences become ‘too close’. Doing so can be understood as distancing behavior to avoid being potentially overwhelmed by emotionally intense situations. Studies have shown that this is also a common strategy in other professional groups, e.g. in the emergency services (Jonsson & Segesten, 2003, 2004; Regehr, Goldberg, & Hughes, 2002) and nurses (Blomberg & Sahlberg-Blom, 2007; Goldblatt, 2009; Wallerstedt, Benzein, & Andershed, 2011). This helpful coping mechanism must be distinguished from emotional detachment in the context of burnout. Furthermore, burnout is connected to lack of enthusiasm, depersonalization, hopelessness, lower self-esteem, cynicism, and inefficiency (Alexander, 1999; Maslach, 2003). Sustained negative experiences, which do not have to be traumatic, are a prerequisite for burnout (Tracy, 2012).

In addition, many volunteers reported spending some time alone after emergency responses. This involved engaging in recreational activities (e.g. listening to music, cooking, walking) as well as reflecting on the emergency response. Emergency response reports are a mandatory organizational requirement and many volunteers stated to intuitively use this compulsory activity as a reflective processing tool. The volunteers described that the clear procedural framework of an emergency response assignment with a defined beginning (getting ready with their response partner) and end (writing the response report) helped them cope with and close the respective response case. This approach is evocative of narrative techniques used in trauma therapy which aim at developing a narrative of the experienced situation. (Nikendei, Greinacher, & Sack, 2017).

The volunteers also reported using different rituals (e.g. wearing a special uniform during or taking a talisman to emergency responses) to facilitate shifting between their professional role and their everyday life. Especially expert volunteers highlighted the importance of only participating in emergency responses when feeling mentally and physically stable. As part of the psychosocial emergency care of the German Red Cross, the volunteers can decide flexibly when they want to participate in emergency responses. Furthermore, they can exclude certain specific emergency responses, if they feel unable to cope with them, e.g. deaths of children. A quantitative study on the psychological burden of volunteers in emergency psychosocial care revealed a secure attachment style, a good structural level, and a sense of coherence as well as mindfulness as key resilience factors in this group (Greinache et al., 2019b).

### Positive Impact of Psychosocial Emergency Care Work

In addition, volunteers reported on changes in their own lives because of their volunteer work. Feeling more concern regarding their personal safety and their families’ well-being, some of the volunteers sought to achieve greater safety by taking out long-term care insurance or taking more care when driving. Perhaps this could point to vicarious traumatization as outlined above. On the other hand, many volunteers experienced as sense of greater contentedness as well as feeling a greater humility with regard to their own lives. They expressed their appreciation and recognition for their colleagues and highlighted a strong sense of experienced work-related group solidarity. Overall, they expressed their deep conviction regarding the meaningfulness of their work. These positive changes can be conceptualized as posttraumatic growth - ‘the experience of individuals whose development, at least in some areas, has surpassed what was present before the struggle with crises occurred’ (Tedeschi & Calhoun, 2004). This may be reflected in a higher appreciation of life, more meaningful relationships, a sense of personal strength, changed priorities, and an enriched existential and spiritual life.

### Practical Implications

Following the study’s results, implications for emergency response practice should be discussed. Although subclinical, all volunteers reported emotional and physical reactions to the emergency responses. Therefore, these findings should be considered in conjunction with emergency psychosocial care work. Individuals experiencing high psychological burden, reduced capacity for reflection, or feel that they are trying to solve personal problems by supporting people in crisis should receive support from the organizations. Potential volunteers should be fully informed about the work in emergency mental health care, including the risks and side effects, right from the beginning of the introduction and selection process. Candidates should be asked about their past and present personal exposure to stress and their respective coping strategies. It should be determined whether applicants are capable of communicating their situation and seeking help in difficult situations. Personal resources, such as an active social environment and leisure activities, should also be addressed. Applicants should be asked about their personal motivations for volunteering to clarify what candidates hope to achieve or gain by working in emergency care. In particular, candidates should be asked about previous experience and whether they have a realistic picture of the work involved. Should this interview process raise any concerns, organizations should discuss whether the work is suitable for them and what kind of support they need with their volunteers. If necessary, volunteers should be given contact details of possible external contacts, such as counselling or psychotherapy. We recommend evaluating one’s own personal health before embarking on, though not unrewarding, potentially highly stressful emergency psychosocial care work. If volunteers are experiencing high levels of stress, they should be permitted to be exempt from emergency responses for a certain period of time. Since individual suitability for this work can change over time (Powell et al., 2014), psychological levels of stress should be assessed regularly, e.g. regular staff interviews. In cases where supervisors are concerned that volunteers may be developing symptoms following emergency responses, they need to communicate this as part of their responsibility as caregivers. If life circumstances change, it is also important to re-evaluate which emergency responses are appropriate, e.g. new parents may fail in emotionally distancing themselves from emergency responses in which infants have died. Constellations of this kind require special preparation, reflection, and re-evaluation.

Social support is one of the most important protective resources against secondary traumatization. In selection procedures, organizations should ensure that candidates have a healthy social network. Providing volunteers with support at the organizational level is also helpful and organizations should encourage communication about mental health issues. Volunteers should feel free to talk about their feelings und how their work affects them. Organizations need to establish frameworks facilitating communication and discourse, about emergency responses and related reactions, e.g. team recreational evenings, regular inter- and supervision, as well as debriefing sessions. In addition, a specific contact person should be defined to whom volunteers may turn in confidence when necessary. Emergency responses should always be worked as a team allowing the participants to exchange information directly. The volunteers’ work-related psychological needs to be taken seriously and, where necessary, addressed in follow-up work, such as supervision. However, such measures are intended to support volunteers and should not be perceived as punishment for inaptitude. Regular and mandatory supervision is recommended to enable the discussion of emergency responses and team conflicts as well as giving volunteers the opportunity to share their coping strategies, e.g. helpful rituals. To encourage an open atmosphere, supervisions should be led by external supervisors. Furthermore, supervision not only encourages the discussion of difficulties and stress factors, but also promotes active problem solving, self-reflection and capacity building (Wyche et al., 2011). Preferably, invited supervisors are not only well grounded in the field of psychology (e.g. psychological burden), but are also experienced in emergency psychosocial care (Burns et al., 2008).

### Limitations

As interviews were exclusively conducted with German Red Cross Baden-Württemberg volunteers, results cannot be generalized. This study is further limited by the variance in the type and frequency of emergency responses as well as in the format and frequency of supervision.

## Conclusion

In previous studies, first responders showed relatively low levels of psychological stress such as secondary traumatization, anxiety and depression. In this study, we interviewed volunteers in psychosocial emergency care - a subgroup of first responders - in order to better understand their burdens and resources. It turned out that the study participants showed emotional and physical reactions to their emergency responses, but these can be considered normal reactions to severe stress. The volunteers described social support as particularly helpful. Further strategies were to keep an inner distance from the events during emergency responses, to cultivate rituals, and to do good to oneself in the emergency responses. Organizations that provide psychosocial emergency care should consider several things. Because participants in the study reported stress from emergency responses, care should be taken to ensure that volunteers bring sufficient personal resources with them when they begin training as psychosocial emergency responders. If volunteers report increased stress, they should temporarily not undertake emergency responses until they have stabilized. The organizations can also strengthen their staff by promoting social support. This can be done through group evenings, team meetings or supervision.

## Data Availability

The datasets used and/or analyzed during the current study are available from the corresponding author on reasonable request.

## Abbreviations

EXP: experienced volunteers who had completed their training and had participated in regular emergency responses
PRE-NOV: novice volunteers just starting their training who had not yet participated in any emergency responses
POST-NOV: novice volunteers who had just completed their training and had accompanied experienced volunteers to emergency responses
